# Relating Sub-Surface Ice Features to Physiological Stress in a Climate Sensitive Mammal, the American Pika (*Ochotona princeps*)

**DOI:** 10.1371/journal.pone.0119327

**Published:** 2015-03-24

**Authors:** Jennifer L. Wilkening, Chris Ray, Johanna Varner

**Affiliations:** 1 Department of Ecology and Evolutionary Biology, University of Colorado, Boulder, Colorado, United States of America; 2 Department of Ecology and Evolutionary Biology and Institute for Arctic and Alpine Research, University of Colorado, Boulder, Colorado, United States of America; 3 Department of Biology, University of Utah, Salt Lake City, Utah, United States of America; The Ohio State University, UNITED STATES

## Abstract

The American pika (*Ochotona princeps*) is considered a sentinel species for detecting ecological effects of climate change. Pikas are declining within a large portion of their range, and ongoing research suggests loss of sub-surface ice as a mechanism. However, no studies have demonstrated physiological responses of pikas to sub-surface ice features. Here we present the first analysis of physiological stress in pikas living in and adjacent to habitats underlain by ice. Fresh fecal samples were collected non-invasively from two adjacent sites in the Rocky Mountains (one with sub-surface ice and one without) and analyzed for glucocorticoid metabolites (GCM). We also measured sub-surface microclimates in each habitat. Results indicate lower GCM concentration in sites with sub-surface ice, suggesting that pikas are less stressed in favorable microclimates resulting from sub-surface ice features. GCM response was well predicted by habitat characteristics associated with sub-surface ice features, such as lower mean summer temperatures. These results suggest that pikas inhabiting areas without sub-surface ice features are experiencing higher levels of physiological stress and may be more susceptible to changing climates. Although post-deposition environmental effects can confound analyses based on fecal GCM, we found no evidence for such effects in this study. Sub-surface ice features are key to water cycling and storage and will likely represent an increasingly important component of water resources in a warming climate. Fecal samples collected from additional watersheds as part of current pika monitoring programs could be used to further characterize relationships between pika stress and sub-surface ice features.

## Introduction

Climate change in the form of rising temperatures, changing precipitation patterns and increased frequency of extreme weather events is occurring at an unprecedented rate [1]. Shifting climates are affecting species distributions, phenology, and physiology worldwide [2]-[4]. Increased atmospheric CO_2_ has also resulted in changes in plant productivity and plant-herbivore interactions [5], [6].

Climate change is also likely to have profound impacts on ecological systems and the services they provide. For example, much of the world’s human population depends on water resources that originate in alpine ecosystems, and up to 80% of the planet’s fresh surface water comes from high elevation watersheds [7]. These mountainous and highland regions are often referred to as the world’s natural “water towers”, and evidence suggests that warming may occur more rapidly at these higher elevation locations [8]-[11]. Climate warming has already forced a global retreat of glaciers [12] as well as declines in the number of winter days with frost [13] and the duration of snowpack at low and middle elevations [14]. As a result, water derived from snowmelt in the US Pacific Northwest has decreased by 20% since 1950 [15]. Similar declines around the world will have far reaching consequences for both humans and wildlife [1].

One important contributor to these alpine water resources is rock glaciers, or permafrost insulated by a covering of fragmented rock. Rock glaciers and associated rock-ice features (RIFs) typically occur in arctic and alpine landscapes characterized by cold temperatures, low humidity, and the presence of talus or broken rock [16]. Most studies documenting the decline of glaciers worldwide have measured retreats of surface ice, which tends to melt faster than rock glaciers. Located beneath an insulating layer of rock fragments (talus), rock glaciers are less affected than glaciers by rising air temperatures [15]. As climate change depletes surface water-storage features (i.e., glaciers and snowpack) [15]-[17], RIFs will become increasingly important as water reservoirs [18].

The hydrologic contribution of RIFs remains understudied [19] despite the rising need to understand their role in water cycling [20], in part because RIFs can be extremely difficult to detect and are often overlooked in the environment [19]. Nevertheless, RIFs have long been associated with one periglacial species [21]-[23], the American pika (*Ochotona princeps*). Pikas are cold-adapted members of the rabbit order that require cool microclimates and talus habitat [21]. As a result of recent climate-mediated population declines, pikas have become widely considered a sentinel species for detecting ecological effects of climate change [21]-[29]. Here, we consider the physiological implications of the pika’s association with RIFs. The sub-surface habitats in talus associated with RIFs tend to be relatively cool in the summer and warm in the winter [30], climatic conditions that have been positively related to pika persistence [31]-[33]. Thus we hypothesized that pikas living in RIFs would experience lower levels of physiological stress.

Analyses of stress hormone metabolites in fecal material are increasingly employed to evaluate the health and fitness of sensitive animal populations [34]-[36], and these techniques have recently been validated for pikas [37]. Because samples can be collected without disturbing or endangering the animal, these measurements are not biased by capture-induced stress, and reflect an average level of circulating stress hormones. Therefore, analysis of fecal samples can provide the most precise estimate of the current endocrine condition of an animal [34], [38], [39]. Although fecal stress hormone metabolites have been increasingly used as proxies of animal health, few studies account for the potential influence of environmental factors when using fecal samples collected non-invasively. Environmental conditions such as temperature or humidity can alter microbial activity, which can result in decomposition of steroid metabolites and biased measurements of metabolite concentration [40]-[43].

In this study, our first objective was to examine the post-deposition effects of varying environmental conditions on stress hormone metabolite concentration (glucocorticoid metabolite; GCM) in pika fecal pellets. By comparing GCM concentration measured in pika fecal pellets that were placed outside in timed exposure trials, we tested whether environmental conditions directly alter GCM concentrations measured in samples. Our second objective was to use GCM concentration, measured non-invasively from fecal pellets, as a metric of stress in pikas living in habitats with and without RIFs. If habitats underlain by ice promote pika health, then we hypothesized that GCM concentration would be lower in samples collected from within RIFs. To further characterize this relationship, we modeled GCM concentrations in pika scat as a function of microclimatic conditions measured *in situ* over the previous two years.

## Materials and Methods

### Study area

This research was conducted in the US Rocky Mountains at five study sites: Niwot Ridge Long Term Ecological Research Site (NWT), Green Lakes Valley Watershed (GLVW), Brainard Lake Recreation Area (BLRA), and Rocky Mountain National Park (RMNP) in Colorado, and Emerald Lake (EL) in Montana (Table 1). All sites comprised numerous patches of typical pika habitat, characterized by large regions of broken rock (talus) interspersed with alpine meadows ranging in elevation from approximately 2500 to 3700 m.

**Table 1:** Site name and abbreviation as used throughout the study.

Site Name	Site Abbreviation	Mean Latitude	Mean Longitude	Elevation range (m)
Niwot Ridge LTER	NWT	40.06	105.60	3587–3625
Green Lakes Valley Watershed	GLVW	40.05	105.62	3458–3779
Brainard Lake Recreation Area	BLRA	40.07	105.59	3300–3326
Rocky Mountain National Park	RMNP	40.40	105.67	3246–3313
Emerald Lake	EL	45.41	110.93	2748–2846

Mean latitude and longitude refer to the average location within each site where samples were collected or exposed. Elevation range gives the range of elevations of locations within each site where samples were collected or exposed.

### Objective 1: Exposure trials

To obtain fecal samples for the exposure trials in objective 1, pikas at NWT and BLRA were live trapped and fresh fecal pellets were collected only from adult female pikas (to control for observed differences in GCM concentration resulting from gender and age) during summer 2012. Light anesthesia (inhalant anesthetic, Isoflurane) was maintained throughout the 20-minute handling process and all efforts were made to minimize suffering. After handling, pikas were released back into their home territories. Trapping and sampling procedures were reviewed and authorized by Colorado Parks and Wildlife (license no. TR2014) and procedures followed those approved by the University of Colorado-Boulder Institutional Animal Care and Use Committee (protocol 1104.06). Fecal samples were kept on ice in the field and transferred within 12 h to a −20°C freezer. Pellets collected from 11 different individuals were then pooled, mixed and divided at random into three controls plus 12 samples slated for exposure. Each exposure sample consisted of approximately 15 pellets placed inside a modified plastic food-storage container (9 cm × 9 cm × 12 cm) with a detachable lid and mesh sides. These “exposure boxes” were then semi-buried within the talus environment during August 2012 at 3 different locations within each of four sites: NWT, BLRA, RMNP and EL. Placement of exposure boxes mimicked the location of naturally occurring pika latrines by shielding pellets from direct sunlight and rain. Boxes were placed only in currently occupied pika territories, adjacent to fresh fecal piles. After two weeks, exposure boxes were collected from all sites, and fecal samples were transferred to the lab for analysis. Control samples were maintained in the -20°C freezer during the entire exposure period and GCM analysis was performed on all samples at the same time.

GCM extraction and analysis proceeded according to protocols previously validated for pikas [37]. Briefly, fecal samples were lyophilized and ground into powder using a mortar and pestle. We then followed the steroid solid extraction protocol provided by Arbor Assay Design, Inc. Comparative analysis of GCM levels in samples was conducted using a commercially available Corticosterone Enzyme Immunoassay Kit (Arbor Assay Design, Inc., Ann Arbor, MI; cat. no. K014-H1). During each assay, we ran extracted samples in triplicate alongside a standard curve of seven known concentrations of corticosterone (5000, 2500, 1250, 625, 312.50, 156.25, 78.125 pg/ml.) Values for each extracted sample were generated using a micro plate reader (BioTek Microplate Reader Synergy HT; 2005 Biotek Industries, Inc.) and Gen 5 1.11 Data Analysis software. Intra-assay coefficients of variation were less than 10% and inter-assay coefficients of variation were less than 15%. Final concentrations of fecal GCM were expressed as ng GCM/g dry feces (these data and all additional data described are uploaded as S1 Table).

One-way ANOVA was used to test for differences in GCM concentration among control samples and exposed samples at each site. Prior to analysis, data were checked for outliers, normal probability plots were examined, and a Shapiro-Wilks statistic was calculated to test for normality. All statistical analyses were conducted using R 3.0.1 (R Core Team 2013) and significance was assessed at α = 0.05.

### Objective 2: Sample and temperature data collection

To compare GCM concentration across habitat types in objective 2, we collected feces from two adjacent sites: one with RIFs (GLVW) and one without RIFs (NWT). These areas are situated at the northern edge of the Front Range, near Boulder, Colorado and comprise an elevational range of 3200–4000 meters (Table 1, S1 Fig.). The region is characterized by low temperatures throughout the year and receives most of its precipitation in the form of winter and spring snowfall. The GLVW consists of two alpine catchments, and provides approximately 40% of the water supply for the City of Boulder. It also has persistent rock glaciers and other sub-surface ice features [44]. NWT has been the location of hydrological and snow studies since 1971 [45], [46]. In contrast to GLVW, permafrost and similar RIFs have not been found at NWT since 2008. Recent research documents the absence of permafrost at NWT, suggesting that previously designated permafrost areas have melted over time and/or were initially overestimated [47]-[49].

Pika scat is easy to identify and was abundant at both GLVW and NWT. Fresh fecal samples were collected from territories occupied by adult pikas during summer 2011–2013 at both sites (N = 34 samples from GLVW, 30 from NWT). Pikas maintain established latrines within individual territories, and fecal samples were identified as fresh by color, consistency, and relative position. Samples were immediately put on ice after collection, and transferred to -20°C prior to analysis. Care was taken at both sites to collect samples from locations of varying aspect and elevation. Extraction and estimation of GCM concentration from fecal samples followed procedures described above.

We also characterized microclimates at each site, which might contribute to observed patterns of physiological stress. In models designed to predict patterns of recent pika declines, the relative importance of climatic factors has risen dramatically over the past decade [32]. Pikas have a narrow thermal tolerance; when ambient temperatures increase, they reduce their activity levels and shed heat passively by retiring to cooler microclimates [50], [51]. In addition, pikas also appear to be sensitive to cold stress during winter, due to reduced snowpack and consequent loss of insulation from sub-freezing temperatures [29], [31], [32], [52]. At each site, we placed 18–20 temperature data loggers (DS1921G, Thermochron iButtons, Maxim Integrated Products, Sunnyvale, CA) inside the talus habitat, approximately 0.5–1.0 meters below the surface. These data loggers recorded temperatures of pika relevant microclimates every four hours for two years (2011–2013).

### Data analysis, response and predictor variables

We applied F-tests to assess the equality of variance between samples, and Welch’s t-test to identify overall differences in GCM concentration between samples collected at GLVW and NWT. Prior to analysis, data were checked for outliers, normal probability plots were examined, and a Shapiro-Wilks statistic was calculated to test for normality. All statistical analyses were conducted using R 3.0.1 (R Core Team 2013) and significance was assessed at α = 0.05.

For models of GCM concentration, candidate predictor variables represented potential indicators of chronic and acute stress identified in previous studies of the American pika [31]-[33], [53], [54]. These included: elevation, average summer temperature, number of days below negative 10°C, potential solar gain, and summer diurnal temperature range. Predictors were abbreviated as:

ELEV = elevation in meters,
AST = average summer temperature June-September,
DB-10 = number of days below negative 10°C,
PSG = potential solar gain, and
SUMDTR = average summer diurnal temperature range June-September.

Elevation (ELEV) was recorded in meters at each fecal sampling location as a proxy for both chronic heat and cold stress. Average summer temperature (AST) represents a measure of chronic heat stress, and was calculated by averaging all sub-surface temperature measurements during June-September. Number of days below 10°C (DB-10) is an indicator of acute cold stress, and this was measured by summing the total number of days throughout the year that experienced sub-surface temperatures below this threshold. Potential solar gain (PSG) exemplifies how slope and aspect interact to create individualized micro-climatic conditions leading to chronic heat or cold stress. Aspect and slope were recorded at each fecal sampling location using a compass and inclinometer. PSG was then estimated as sine (slope) x cosine (aspect), which produced values ranging from -1 to 1: steeper, north facing slopes have larger positive values, and steeper, south facing slopes have larger negative values [54]. Summer diurnal temperature range (SUMDTR) was considered an indicator of both acute and chronic heat stress. This variable has not been considered previously in analyses related to pikas and climate change; however, climate change is affecting diurnal temperature range, and other alpine species appear to be responding to these increased fluctuations [55]. Diurnal temperature range measurements may differ between seasons [56], [57] and we used only a summer value to test for physiological constraints pikas may experience during the summer foraging season. Summer diurnal temperature range was obtained by averaging the daily difference between sub-surface temperature maxima and minima measured during June-September.

### Timing of fecal sample and temperature data collection

GCM response was measured in fecal samples collected from GLVW during 2011–2013 and from NWT during 2012–2013. Time-averaged temperature metrics were obtained from data loggers continuously in place at both sites from 2011–2013. Fecal samples were not always available within the immediate vicinity of data loggers, so a distance matrix was used to associate fecal samples with distance-weighted average temperature metrics. For example, the distance-weighted average summer temperature over 2011–2013 at sampling location *i* was calculated as AST_*i*_ = ∑_*j*_ AST_*j*_ (1/d_*ij*_ / [∑_*j*_ 1/d_*ij*_]), where d_*ij*_ was the distance between fecal sample *i* and data logger *j*, for *j* = 1 to *n* data loggers at the study site. The average minimum distance between samples and data loggers was 33.23 meters (SD = 25.10) at NWT and 233.41 meters (SD = 193.31) at GLVW. This difference reflects the smaller size of the NWT study area and higher density of data loggers available at that site (placed for other studies). Although GLVW encompassed a larger area and data logger density was lower at that site, physiographic variables (such as elevation) varied little between sampling locations within GLVW (Table 1).

### Linear mixed effects models

Fecal GCM concentration, a validated indicator of physiological stress [37], was used as a response variable in linear mixed-effects models. Mixed-effects models were used to address repeated sampling from similar locations [58]. Models were developed and then compared using an information-theoretic framework. We evaluated the relative support for each predictor variable within and between sampling areas using 34 candidate models (S2 Table). This model set represents alternative stressors (i.e., predictor variables), as well as stressors working in combination. Several predictor variables were highly correlated (Spearman’s r > 0.5), some within sites and some across the two-site dataset. To maximize the number of models considered while avoiding collinearity, we examined GCM response in three separate analyses: 1) within GLVW, 2) within NWT, and 3) across both sites. Models based on combinations of highly correlated predictors (Spearman’s r > 0.5) were not considered within these 3 model sets (S3 Table).

The relative support for each model within each analysis was calculated using an information criterion (AIC) [59]. We considered models with an AIC score 0–2 units higher than the lowest observed score to have similar and strong support, while models with a score 4 or more units higher had little support. Model averaging and the ranking of predictor variables were performed using the R package MuMIn [60] for the analysis of a global model including all 5 predictors and no interaction effects.

Models were fit using *lme4* [61] in R 3.1.0 (R Development Core Team 2014). Models contained one or more predictor variables (ELEV, AST, DB-10, PSG, SUMDTR) as fixed effects. Site (analysis 3) and year were treated as random effects on model intercept and slope(s). Fixed effects differed by model, but random effects were the same for every model within each analysis. Since only fixed effects differed among models, maximum likelihood (rather than restricted maximum likelihood) was used to estimate coefficients and compare these nested models [62]. Interaction terms were omitted if not significant (p-value > 0.10).

In addition to model comparison using AIC, pseudo-R^2^ values were calculated for each model as a metric of goodness-of-fit. Using methods specified for linear mixed-effects models [63], we computed both a marginal R^2^ (i.e., amount of variance explained by the fixed effects) and a conditional R^2^ (i.e., amount of variance explained by the fixed and random effects in combination). Models were ranked according to ΔAIC value, rather than R^2^, to ensure an appropriate penalty for each predictor variable.

We examined residuals of the best model in each analysis in two ways. Residual plots were visually inspected for deviations from homoscedasticity and normality. To check for spatial auto-correlation in model residuals, we used the *ape* library (v. 3.0–11), [64] to calculate Moran’s I coefficients.

## Results

### Exposure trials

**Fig 1:**
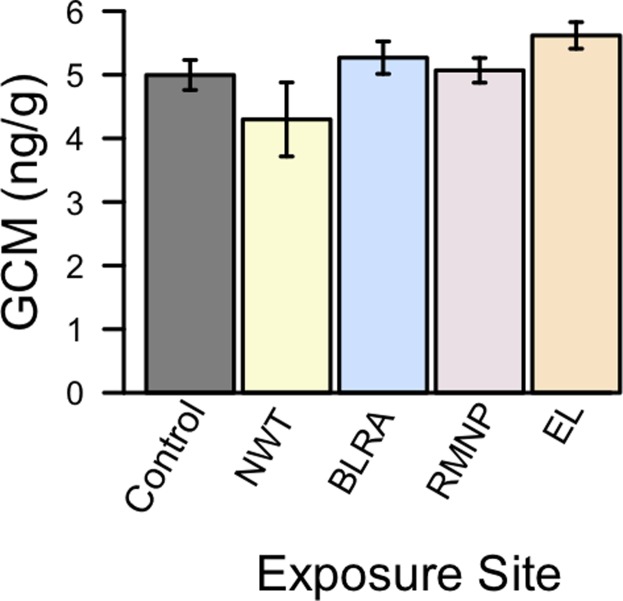
Measured GCM concentration in controls and samples exposed at sites in the Rocky Mountains. GCM concentration is expressed in nanograms/gram. Exposure sites were Niwot Ridge LTER (NWT), Brainard Lake Recreation Area (BLRA), and Rocky Mountain National Park (RMNP), Colorado, and Emerald Lake (EL), Montana.

GCM concentration did not differ significantly, either between control and exposure samples or between samples exposed at different sites F_(4,10)_ = 0.73, p-value = 0.59 (Fig. 1). The grand mean GCM concentration across all exposed and control samples was 5.05 (SE = 0.24).

### Temperature comparison between GLVW and NWT

**Fig 2:**
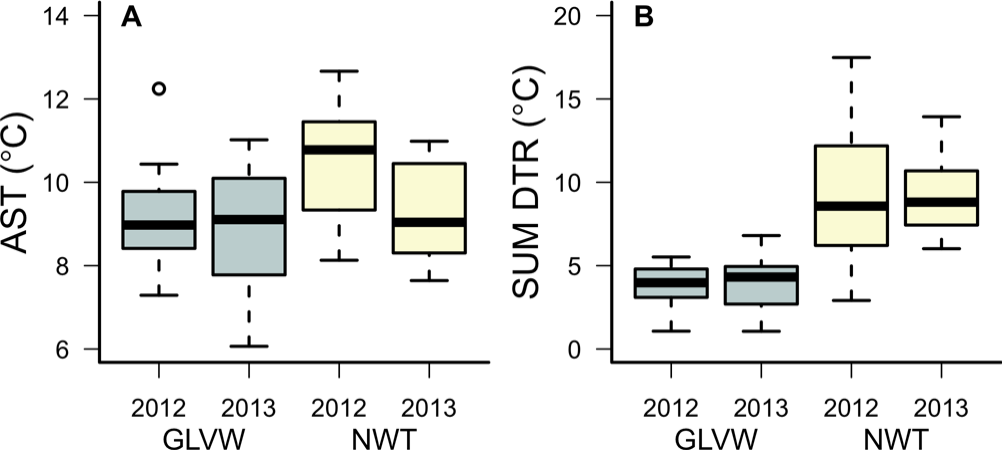
Thermal summaries of temperature variables related to heat stress. Values obtained from sub-surface data loggers placed at Green Lakes Valley Watershed (GLVW) and Niwot Ridge LTER (NWT) during 2011–2013. Boxes depict medians and 25% and 75% quartiles. Whiskers extend through the 95% interquartile range. AST (Average Summer Temperature; Fig. A) corresponds to average temperature during June-September. SUMDTR (Summer Diurnal Temperature Range; Fig. B) corresponds to average diurnal temperature range June-September.

**Fig 3:**
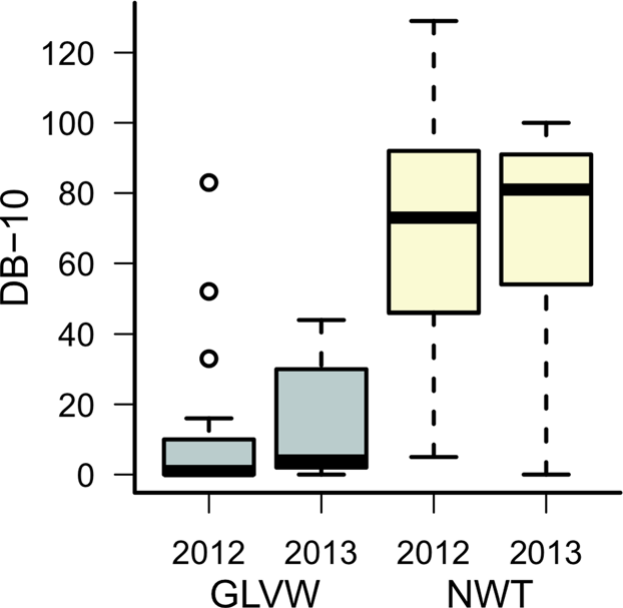
Thermal summaries of a temperature variable related to cold stress. Number of days below -10°C (DB-10) obtained from sub-surface data loggers placed at Green Lakes Valley Watershed (GLVW) and Niwot Ridge LTER (NWT) during 2011–2013. Boxes depict medians and 25% and 75% quartiles. Whiskers extend through the 95% interquartile range.

Thermal summaries revealed sub-surface temperature differences between GLVW (RIFs present) and NWT (RIFs absent). For temperature variables related to heat stress, AST (average summer temperature) was lower at GLVW than at NWT only during 2012 (Fig. 2A). SUMDTR (average summer diurnal temperature range) was lower at GLVW than at NWT during both years (Fig. 2B). Our metric of cold stress, DB-10 (number of days below negative 10°C), was lower at GLVW than at NWT during both years (Fig. 3).

### GCM comparison between GLVW and NWT

**Fig 4:**
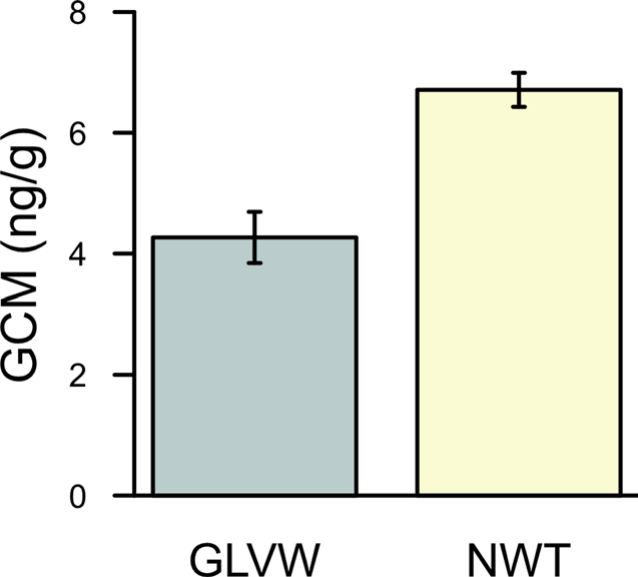
Measured GCM concentration at sites with and without sub-surface ice. Fecal samples were collected during 2011–2013 from a site with RIFs (Green Lakes Valley Watershed, GLVW) and a site without RIFs (Niwot Ridge LTER, NWT). GCM concentration is expressed in nanograms/gram.

An F test revealed a significant difference in the variances of GCM concentrations between GLVW and NWT (F_(33,29)_ = 2.95, p-value < 0.001). GCM concentration was significantly lower in samples from GLVW than from NWT (Fig. 4; Welch’s t-test; p-value < 0.001, df = 54, N = 34 at GLVW, N = 30 at NWT). Average GCM concentration for samples collected during 2011–2013 was 4.27 ng/g (± XXX SE) from GLVW, 6.71 ng/g (± XXX SE) from NWT.

### Mixed effects models

In the first analysis (GLVW alone), ΔAIC indicated that the four best models included one or several of the following predictors: ELEV, AST, DB-10, SUMDTR (Table 2). AIC was lowest for model “ELEV, AST, DB-10” but differed by < 2 for three other models. Two of the top four models (“ELEV, AST, DB-10” and “ELEV, DB-10, SUMDTR”) included an interaction term (“ELEV:DB-10”). All other interaction terms were not significant, and were omitted from the models.

**Table 2:** Relative support for models of pika stress (GCM concentration) in the Green Lakes Valley Watershed.

Model	AIC	ΔAIC	Log(L)	B	Marginal R^2^	Conditional R^2^
ELEV, AST, DB10	156.80	—	-71.40	-0.01, 0.79, -3.79	0.24	0.40
AST	157.99	1.19	-74.99	0.68	0.09	0.28
ELEV, DB10, SUMDTR	158.05	1.25	-72.03	-0.02, -4.79, 1.29	0.21	0.42
AST, DB10	158.67	1.87	-74.33	0.68, -0.03	0.12	0.29
ELEV, AST	159.08	2.28	-74.54	-0.00, 0.63	0.12	0.29
Null	159.92	3.12	-76.96	NA	—	—
AST, PSG	159.96	3.16	-74.98	0.68, 0.14	0.09	0.28
AST, DB10, PSG	160.39	3.59	-74.19	0.70, -0.03, 0.51	0.13	0.31
ELEV	160.55	3.75	-76.27	-0.00	0.03	0.23
DB10	160.78	3.98	-76.39	-0.03	0.03	0.22

Models are ranked in order of increasing AIC values (Akaike’s information criterion). L denotes likelihood. ΔAIC is the difference between the indicated model and the best model (the model with lowest AIC). Unsupported models (ΔAIC > 4) are not shown. Marginal R^2^ represents the amount of variance explained by fixed effects alone, while conditional R^2^ represents the amount of variance explained by fixed and random effects in combination.

In the second analysis (NWT alone), AST and DB-10 again appeared in the two models with highest support (Table 3). No interaction of AST and DB-10 was supported. The highest marginal and conditional R^2^ values were found in models “AST, DB10” and “AST, PSG”.

**Table 3:** Relative support for models of pika stress (GCM concentration) at the Niwot Ridge LTER site (NWT).

Model	AIC	ΔAIC	Log (L)	B	Marginal R^2^	Conditional R^2^
DB10	101.45	—	-46.72	-0.04	0.35	0.35
AST, DB10	102.06	0.61	-46.03	0.52, -0.04	0.38	0.38
AST, PSG	104.23	2.78	-46.12	2.79, -97.17	0.38	0.38
Null	111.99	10.54	-53.00	NA	—	—

Definitions are as in Table 2.

In analysis 3 (including data from both sites), the best models were based on AST and ELEV (Table 4). No interaction of AST and ELEV was supported. DB-10, PSG and SUMDTER appeared in models with weak support. Akaike weights indicated that AST (.85) was the strongest predictor, followed by DB10 (0.69; Table 5).

**Table 4:** Relative support for models of pika stress (GCM concentration) across both study sites (Green Lakes Valley Watershed and Niwot Ridge LTER).

Model	AIC	ΔAIC	Log (L)	B	Marginal R^2^	Conditional R^2^
AST	275.91	—	-132.95	0.63	0.08	0.33
ELEV, AST	277.26	1.35	-132.63	-0.00, 0.60	0.09	0.34
DB10	278.26	2.35	-134.13	-0.02	0.08	0.58
Null	279.28	3.37	-135.64	NA	—	—
PSG, SUMDTR	279.41	3.50	-132.70	-4.63, 0.43	0.18	0.33

Definitions are as in Table 2.

**Table 5:** Relative support for predictors of pika stress.

Predictor	Akaike weight
AST	0.85
DB10	0.69
ELEV	0.37
PSG	0.37
SUMDTR	0.26

Akaike weights were calculated across all possible linear mixed models of data from both study sites, where each model included only direct effects based on 1–5 of the 5 predictor variables.

Across all three analyses, ELEV was negatively correlated with GCM concentration, while AST, SUMDTR and DB-10 were all positively correlated with GCM concentration. Moran’s *I* statistic indicated no spatial autocorrelation in residuals from the best models of data from GLVW (p-value = 0.44), NWT (p-value = 0.96) or both sites combined (p-value = 0.31).

## Discussion

Although fecal stress hormone metabolites are commonly used to assess animal health and fitness, few studies account for the influence of environmental exposure on GCM measurements of samples collected non-invasively (i.e., after deposition). We found that exposure of fecal samples to microclimatic conditions relevant to pikas across our study region did not alter GCM concentration. Major factors known to increase or decrease GCM concentration in fecal samples include fluctuations in temperature and precipitation conditions [40]-[43]. In our study, all samples were exposed during a brief period (August 2012) when microclimatic conditions might not have varied considerably across our study sites. We suggest that non-invasive sample collection can be used to reliably compare relative stress hormone metabolite concentration in pika scat across this region within a short sampling period, but we caution against comparisons at larger spatial and temporal scales. The effect of environment on GCM concentration in exposed fecal samples requires further investigation within and between more climatically variable regions, before we can conclude that environmental factors do not measurably influence GCM concentration.

Having established that GCM concentrations were not affected by exposure within our region and sampling period, we then compared physiological stress in pikas (as indicated by fecal GCM concentration) and found that stress was significantly predicted by climate variation within and between sites. Specifically, GCM concentration was significantly higher in pika territories with higher summer temperatures (AST, SUMDTR) and a higher frequency of extremely cold days in winter. Cold sub-surface temperatures in winter are likely due to recent reductions in snow cover [65]. These results are consistent with other studies of pika population occupancy and persistence [31]-[33], [53], [54], [66], [67] adding strong support for the hypothesis that recent pika population declines are due to individuals being physiologically impacted by heat and/or cold stress. Although the relationship between individual fitness and physiological stress in pikas is still under investigation, analyses have identified negative correlations between fecal GCM concentration and annual survival in other species [68]-[70]. Thus, it is likely that the detection of high GCM concentration can be used to identify populations in the very early stages of decline. By establishing a link between specific climatic variables and physiological stress in pikas, our results also provide further evidence in support of pikas as an indicator species of climatic change.

Additionally, our results support the importance of RIFs in maintaining suitable microclimates for pikas. Overall GCM concentration was significantly lower in pikas living among the RIFs of GLVW than in pikas living without RIFs at NWT. Other studies have also indicated that the presence of RIFs appears to improve the suitability of pika habitat. For example, in an assessment of 421 pika sites in mountainous regions of the southwestern USA, 83% of pika-occurrence sites occurred in RIF landforms [30], suggesting that pikas either preferentially select these areas or fail to persist outside of them. Similar to other studies [30], our data suggest that microclimates associated with RIFs are moderate across the year (i.e., cool in the summer and warm in the winter). Although we recognize the limitations of this study in that our preliminary results derive from only two locations within one watershed, our techniques provide a basis for replication in additional watersheds.

In addition to microclimate, RIFs may also mediate pika habitat suitability through vegetation quantity and quality. Specifically, dense wetland vegetation often occurs adjacent to RIF areas, and plant diversity is higher in these areas than in non-wetland alpine habitats [30]. Water availability is also higher near RIFs; thus, vegetation growing near RIFs could have higher water content, a feature for which pikas select when foraging [71]. Metrics of forb cover and similar vegetation-related characteristics have already been shown to predict pika population persistence [33], [67]. These RIF-associated meadows could therefore potentially have higher forage value for pikas. Future studies could include soil moisture measurements, in order to further define the relationship between environmental characteristics of RIFs and pika stress.

Variation in GCM concentration has been explained by differences in diet in some species. In addition to physiological stress resulting from lower quality diets, dietary fiber may also affect fecal mass and gut passage time, both of which could influence final GCM concentration [72], [73]. It is therefore possible that differences in GCM concentration could result from differences in forage between RIF and non-RIF habitats. However, our research related to a multi-regional assessment of fecal GCM revealed no significant influence of vegetation type on GCM concentration measured in pika feces, despite considerable differences in regional vegetation communities across our sample collection area [74]. Furthermore, although we did not quantify vegetation at sampling sites, the adjacent sites used in this study (GLVW and NWT) appeared to support similar vegetation communities. Given that pikas are generalist herbivores and tend to consume plant species in the summer according to relative abundance [75], [76], pika diet seems unlikely to have differed enough between these sites to artificially influence GCM concentration.

Other factors, including predator density and pika population density, have also been shown to positively correlate with GCM measurements across species. Pikas are preyed upon by weasels [77], and it is possible that weasel abundance could differ between sites with RIFs and those without; however, no evidence exists to suggest that weasel abundances are correlated with the presence of RIFs or that they differ between our adjacent study sites. Pika population density could also affect GCM measurements through social interactions, increased aggression, or resource competition. Although we did not directly measure pika population density at either site, this variable did not appear to differ between GLVW and NWT. However, annual survival at NWT is relatively low [74]. If GLVW supports superior habitat and serves as a source for the population at NWT, then resident individuals in GLVW may experience lower levels of stress than immigrant individuals at NWT. Future research will be necessary to mechanistically disentangle the effects of microclimate, diet, population density, and predator abundance on stress levels measured in individual animals, and which of these stressors may be related to the presence of RIFs.

Most efforts to identify the effects of climate change on pikas and other species have relied on the establishment of relationships between climatic characteristics and species distribution. Many species expand or contract their ranges in response to changing temperatures, and significant global bioindicators have been developed based upon past and current observations. For example, the presence of palms has traditionally been used in the paleo-botanical literature to identify warmer climates [78]. Due to shorter and warmer winters, palms have recently spread northward as far as southern Switzerland, and have been identified as a bioindicator of current climate change in Europe [79]. Similarly, a rapid increase in vascular plant abundance in response to warmer temperatures has been interpreted as a bioindicator of climate change in Antarctica [80]. Bioindicators can also identify shifting precipitation patterns, another impact of climate change. The larvae of some invertebrates, such as *Diptera*, decline in abundance with decreased levels of soil moisture [81], [82] and the absence of these organisms has been used to indicate climate change.

These examples rely upon distributional shifts at the population level to indicate the extent of climate change. In contrast, studies identifying indicators of change based upon physiological response of individual organisms are far less common, though they could be extremely valuable as earlier and more sensitive indicators of change. These studies are becoming more feasible as the relationship between physiological stress and climatic characteristics is documented in more species. For example, higher GCM concentrations have been measured in several species in response to reduced precipitation (e.g., Koalas [83], African elephants [84], and spider monkeys [85]). Similarly, temperature can significantly influence physiological stress levels, with most examples documenting increased GCM concentrations in response to higher temperatures [86], [87].

Taken together, these studies suggest the utility of using the physiological stress response of organisms such as the pika as bioindicators of habitat quality. Given the relative longevity, mobility and trophic position of most mammals, their physical condition represents a complex set of inputs integrated over space and time. These features make the mammalian stress response an attractive bioindicator of complex and spatially variable changes in habitat quality. Monitoring pikas as an indicator of habitat quality in alpine areas might provide information on attributes of change that cannot be discerned by monitoring abiotic factors alone. Pika monitoring programs are gaining in popularity across the western US, and existing protocols are increasingly incorporating the collection of fresh fecal samples as a standard element of sampling. Although our preliminary results are derived from only one watershed, our techniques can serve as a template for further investigations of the role of RIFs in reducing pika stress. Alpine areas provide excellent proving grounds for the development of climate change bioindicators because they are relatively isolated (and therefore less susceptible to other anthropogenic disturbances) and because changes in climate are expected be more pronounced in these areas [8]-[11]. Pikas are highly detectable, diurnal and charismatic, making them ideal candidates for monitoring as an indicator species. The novel, practical application of small-mammal physiology presented here complements other efforts to model and monitor landscape change in high elevation areas.

## Supporting Information

S1 Fig Map of the Rocky Mountains in the Colorado Front Range, depicting the location of both sampling areas; Green Lakes Valley Watershed (GLVW) with RIFs (solid line) and Niwot Ridge LTER (NWT) without RIFs (dashed line). (TIF)

S1 Table Data used in figures and analyses; column names are as described in the text. (XLSX)

S2 Table Candidate models. Predictors included within each set of candidate models were elevation (ELEV), average summer (June-August) temperature (AST), number of days below negative 10°C (DB-10), potential solar gain (PSG), and average diurnal temperature range during summer (SUMDTR).
(DOCX)

S3 Table Correlation Matrices. Matrices displaying correlations among all predictor variables for Green Lakes Valley Watershed, Niwot Ridge LTER and both sites combined.
(DOCX)
