# Supplementary figures and images for: Relating Sub-Surface Ice Features to Physiological Stress in a Climate Sensitive Mammal, the American Pika (Ochotona princeps)
Source: PLoS One. 2015 Mar 24;10(3):e0119327. doi: 10.1371/journal.pone.0119327 (PMC4372430; doi:10.1371/journal.pone.0119327)

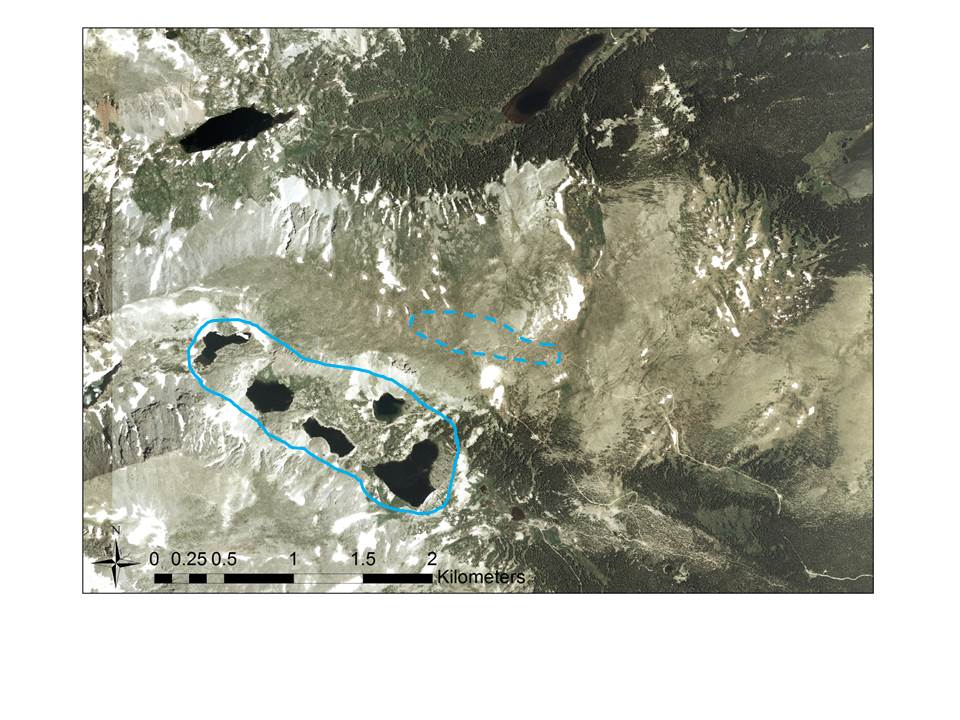

Supplement: S1 Fig — (TIF) [file pone.0119327.s001.tif]
