# Supplementary material for: Relating Sub-Surface Ice Features to Physiological Stress in a Climate Sensitive Mammal, the American Pika (Ochotona princeps)
Source: PLoS One. 2015 Mar 24;10(3):e0119327. doi: 10.1371/journal.pone.0119327 (PMC4372430; doi:10.1371/journal.pone.0119327)
Supplement: S2 Table — Predictors included within each set of candidate models were elevation (ELEV), average summer (June-August) temperature (AST), number of days below negative 10°C (DB-10), potential solar gain (PSG), and average diurnal temperature range during summer (SUMDTR). (DOCX) [file pone.0119327.s003.docx]

**S2 Table. Candidate models.** Predictors in each candidate model include elevation (ELEV), average summer temperature (AST), number of days below negative 10°C (DB-10), potential solar gain (PSG), average diurnal temperature range for summer (SUMDTR).

| **Analysis 1: GLVW alone** |  |
| --- | --- |
| **Model** | **Predictors** |
| 1 | ELEV |
| 2 | ELEV,AST |
| 3 | ELEV,AST, DB-10 |
| 4 | ELEV, DB-10 |
| 5 | ELEV, DB-10, SUMDTR |
| 6 | ELEV, SUMDTR |
| 7 | AST |
| 8 | AST, DB-10 |
| 9 | AST, DB-10, PSG |
| 10 | AST, PSG |
| 11 | DB-10 |
| 12 | DB-10, PSG |
| 13 | DB-10, PSG, SUMDTR |
| 14 | DB-10, SUMDTR |
| 15 | PSG |
| 16 | PSG, SUMDTR |
| 17 | SUMDTR |
| **Analysis 2: NWT alone** |  |
| **Model** | **Predictors** |
| 18 | ELEV |
| 19 | ELEV, AST |
| 20 | AST |
| 21 | AST, DB-10 |
| 22 | AST, PSG |
| 23 | DB-10 |
| 24 | PSG |
| 25 | SUMDTR |
| **Analysis 3: Both Sites combined** |  |
| **Model** | **Predictors** |
| 26 | ELEV |
| 27 | ELEV, AST |
| 28 | ELEV,SUMDTR |
| 29 | AST |
| 30 | DB-10 |
| 31 | DB-10, PSG |
| 32 | PSG |
| 33 | PSG, SUMDTR |
| 34 | SUMDTR |
