# Supplementary material for: Relating Sub-Surface Ice Features to Physiological Stress in a Climate Sensitive Mammal, the American Pika (Ochotona princeps)
Source: PLoS One. 2015 Mar 24;10(3):e0119327. doi: 10.1371/journal.pone.0119327 (PMC4372430; doi:10.1371/journal.pone.0119327)
Supplement: S3 Table — Matrices displaying correlations among all predictor variables for Green Lakes Valley Watershed, Niwot Ridge LTER and both sites combined. (DOCX) [file pone.0119327.s004.docx]

**S3 Table. Correlation Matrices.** Matrices displaying correlations among all predictor variables for Green Lakes Valley Watershed, Niwot Ridge LTER and Both Sites combined.

| **Analysis 1: GLVW** | **ELEV** | **AST** | **DB-10** | **PSG** | **SUMDTR** |
| --- | --- | --- | --- | --- | --- |
| **ELEV** | 1 |  |  |  |  |
| **AST** | -0.15923 | 1 |  |  |  |
| **DB-10** | 0.271024 | -0.00583 | 1 |  |  |
| **PSG** | 0.722987 | -0.13947 | 0.315352 | 1 |  |
| **SUMDTR** | 0.254854 | 0.638894 | 0.098247 | 0.300302 | 1 |

| **Analysis 2: NWT** | **ELEV** | **AST** | **DB-10** | **PSG** | **SUMDTR** |
| --- | --- | --- | --- | --- | --- |
| **ELEV** | 1 |  |  |  |  |
| **AST** | -0.10914 | 1 |  |  |  |
| **DB-10** | -0.64878 | 0.365056 | 1 |  |  |
| **PSG** | 0.525785 | -0.37318 | 0.91084 | 1 |  |
| **SUMDTR** | -0.59191 | 0.552756 | 0.55643 | 0.58389 | 1 |

| **Analysis 3: Both Sites** | **ELEV** | **AST** | **DB-10** | **PSG** | **SUMDTR** |
| --- | --- | --- | --- | --- | --- |
| **ELEV** | 1 |  |  |  |  |
| **AST** | -0.06649 | 1 |  |  |  |
| **DB-10** | 0.532501 | 0.506103 | 1 |  |  |
| **PSG** | 0.61131 | -0.52243 | -0.34405 | 1 |  |
| **SUMDTR** | 0.130451 | 0.650676 | 0.862297 | -0.3143 | 1 |
